# RETRACTED: Argano et al. Protective Effect of Vitamin D Supplementation on COVID-19-Related Intensive Care Hospitalization and Mortality: Definitive Evidence from Meta-Analysis and Trial Sequential Analysis. *Pharmaceuticals* 2023, *16*, 130

**DOI:** 10.3390/ph18111645

**Published:** 2025-10-31

**Authors:** Christiano Argano, Raffaella Mallaci Bocchio, Giuseppe Natoli, Salvatore Scibetta, Marika Lo Monaco, Salvatore Corrao

**Affiliations:** 1 Internal Medicine Department iGR, National Relevance Hospital Trust, ARNAS Civico, Di Cristina e Benfratelli, 90127 Palermo, Italy; raffaellamallacibocchio@gmail.com (R.M.B.); peppenatoli@gmail.com (G.N.); salvusxyz@gmail.com (S.S.); marika.lomonaco@hotmail.it (M.L.M.); s.corrao@tiscali.it (S.C.); 2 Dipartimento di Promozione della Salute, Materno Infantile, Medicina Interna e Specialistica di Eccellenza “G. D’Alessandro”, PROMISE, University of Palermo, 90127 Palermo, Italy

The journal retracts the article “Protective Effect of Vitamin D Supplementation on COVID-19-Related Intensive Care Hospitalization and Mortality: Definitive Evidence from Meta-Analysis and Trial Sequential Analysis” [1], cited above.

Following publication, concerns were brought to the attention of the Editorial Office regarding methodology and the overall validity of the findings presented within this publication [1].

Adhering to our standard procedure, the Editorial Office and Editorial Board conducted an investigation, which confirmed that at least one included study is not a randomized clinical trial (RCT) yet was included in the analysis. Combining such studies could have led to biased conclusions. As a result, the Editorial Board and Editor-in-Chief have lost confidence in the reliability of the findings of this publication [1] and have decided to retract this paper as per MDPI’s retraction policy (https://www.mdpi.com/ethics#_bookmark30).

This retraction was approved by the Editor-in-Chief of the *Pharmaceuticals* journal.

The authors disagree with this retraction.
